# Pathology-Related Influences on the VEM: Three Years' Experience since Implementation of a New Parameter in Phoniatric Voice Diagnostics

**DOI:** 10.1155/2020/5309508

**Published:** 2020-12-21

**Authors:** Constanze Müller, Felix Caffier, Tadeus Nawka, Matthias Müller, Philipp P. Caffier

**Affiliations:** ^1^Department of Audiology and Phoniatrics, Charité-University Medicine Berlin, Campus Charité Mitte, Charitéplatz 1, D-10117 Berlin, Germany; ^2^Department of Pneumology, HELIOS Kliniken Schwerin, Wismarsche Str. 393-397, D-19049 Schwerin, Germany

## Abstract

The vocal extent measure (VEM) represents a new diagnostic tool to express vocal capacity by quantifying the dynamic performance and frequency range of voice range profiles (VRPs). For VEM calculation, the VRP area is multiplied by the quotient of the theoretical perimeter of a circle with equal VRP area and the actual VRP perimeter. Since different diseases affect voice function to varying degrees, pathology-related influences on the VEM should be investigated more detailed in this retrospective study, three years after VEM implementation. Data was obtained in a standardized voice assessment comprising videolaryngostroboscopy, voice handicap index (VHI-9i), and acoustic-aerodynamic analysis with automatic calculation of VEM and dysphonia severity index (DSI). The complete dataset comprised 1030 subjects, from which 994 adults (376 male, 618 female; 18-86 years) were analyzed more detailed. The VEM differed significantly between pathology subgroups (*p* < 0.001) and correlated with the corresponding DSI values. Regarding VHI-9i, the VEM reflected the subjective impairment better than the DSI. We conclude that the VEM proved to be a comprehensible and easy-to-use interval-scaled parameter for objective VRP evaluation in all pathology subgroups. As expected, exclusive consideration of the measured pathology-related influences on the VEM does not allow conclusions regarding the specific underlying diagnosis.

## 1. Introduction

Phoniatric voice diagnostics require sophisticated and highly specialized investigation methods, including objective and subjective measuring procedures [1–3]. Within this multifactorial approach, the recording of a voice range profile (VRP) under standardized conditions represents an established objective and noninvasive tool for the examination of vocal function [4–6]. A stable and straightforward VRP description succeeds when the vocal extent measure (VEM) is applied [7, 8]. This recently developed innovative parameter describes the vocal function as a positive one-dimensional measure, in contrast to the evaluation of dysphonia as a negative criterion by the established multidimensional parameter dysphonia severity index (DSI) [9–11]. The VEM documents the vocal capacity by quantification of the dynamic and frequency range. It is indicated as interval-scaled value usually ranging between 0 and 120, whereas these limits may be exceeded on both sides in severely impaired and exceptionally great voices. Hence, a large vocal capacity results in a high VEM value, conversely, a small VRP is characterized by low VEM. The calculation of this measure is based on the VRP area and perimeter; the detailed mathematical derivation was presented previously [7, 8, 12]. The VEM is recognized as a user-friendly and easily implementable diagnostic instrument. The AVA software [13] computes the VEM automatically after VRP measurement and displays it without additional manual effort or delay.

So far, only a few investigations have been conducted on the evaluation of VEM values. Four studies analyzed the VEM to assess treatment outcomes. Three investigations dealt with benign organic changes of the vocal folds (VF: 61 polyps [12], 37 nodules [14], and 60 edema [15]), another one included 152 patients with various laryngeal pathologies (*n* = 101) and functional dysphonia (*n* = 51) [1]. All of these clinical trials revealed significantly increased VEM values after phonomicrosurgery or conservative treatment. Besides, two other studies evaluated reference ranges of the VEM. The first investigation [8] presented a rough VEM classification deduced from 564 patients with different types of voice disorders, based on the quartiles of the VEM data (≥106: normal vocal capacity; <106 to ≥89: mildly reduced; <89 to ≥64: moderately reduced; <64: severely reduced vocal capacity). The second study [16] introduced gender-specific VEM means (M ± SD) and reference ranges (RR = M ± 1.96 × SD) for young and healthy males (124 ± 13; RR: 99-148) and females (114 ± 13; RR: 88-141). Even though it would simplify the interpretation of vocal complaints in different pathologies and allow for better evaluation of expectable treatment success, more precise and comprehensive data regarding different types of voice disorders as well as healthy voices are not yet available.

Therefore, the purpose of our study was to gather this important information by exploring the influences of pathologic findings on the VEM, three years after the implementation of this parameter in voice diagnostics. It was planned to consider all male and female patients as well as healthy controls in our total study cohort, to examine the effect of specific underlying pathologies compared to other established parameters. Our hypotheses were as follows: the VEM (1) varies between different pathology subgroups, (2) correlates with the corresponding DSI values, and (3) reflects the patients' subjective voice impairment. Furthermore, the VEM should also be investigated with regard to voice usage, gender, and age differences. Based on the findings of the literature, we hypothesized that professional voice use, male gender, and younger age are associated with higher VEM values [7, 8, 14, 16].

## 2. Material and Methods

### 2.1. Participants and Selection Criteria

Three years after the implementation of the VEM [7], a dataset of 1030 consecutive subjects who visited our phoniatric outpatient clinic was analyzed in a clinical retrospective study. Within this 36-month acquisition period, the sample consisted of mainly adult patients with vocal problems, but also of smaller amounts of children, transgender patients, and healthy participants who presented to receive a vocal fitness examination (control group). All children and transgender subjects were excluded from further analysis to avoid distortion of results considering the well-known effects of vocal mutation and maturation [17, 18] as well as conservative or phonosurgical gender-transforming procedures [19, 20] on acoustic and aerodynamic measures. The study was conducted in accordance with the Declaration of Helsinki and approved by the local ethical review board (reference number: EA4/140/10). Selection criteria involved informed consent and completion of the standard phoniatric examination procedures, comprising videolaryngostroboscopy (VLS), acoustic-aerodynamic voice function diagnostics including VRP measurement, DSI, and VEM calculation, as well as subjective self-evaluation using the voice handicap index VHI-9i.

### 2.2. Pathology-Specific Group Assignment

After taking the medical history, a digital VLS followed to assess the etiological diagnosis of the voice disorder and to evaluate the VF vibration, esp. glottal closure, regularity, mucosal wave propagation, and symmetry. It was carried out using a high-resolution rigid videolaryngoscope (10 mm; 70°) with integrated microphone connected to the Endo-STROB control unit (XION Medical, Berlin, Germany). VLS enabled us to divide the cohort into 2 sample groups: structural and functional dysphonia. The diagnoses were categorized and numbered according to the Classification Manual for Voice Disorders by the American Speech Language and Hearing Association (ASHA) [21]. We arranged the study subgroups according to the affected structures and visual pathologies of the VF. Due to the clinical prevalence and importance of malignant laryngeal lesions, we decided to investigate them separately from benign epithelial changes. In these patients, the specific group assignment was based on the postinterventional histopathological result after microlaryngoscopic excision. Healthy participants who passed the vocal fitness examination served as a control group. Finally, the following pathology subgroups were investigated: group I—malignant pathologies, group II—lesions of the lamina propria (e.g., VF nodules, polyps, cysts, and scars; Reinke's edema; and sulcus vocalis), group III—benign changes of the epithelium, group IV—inflammatory changes, group V—neurogenic voice disorders (e.g., unilateral paralyses of the recurrent laryngeal nerve, vagal nerve, or external branch of the superior laryngeal nerve; adductor spasmodic dysphonia; and Parkinson's disease), group VI—functional disorders, and group VII—healthy control group.

### 2.3. Acoustic-Aerodynamic Analysis

Regarding instrument-assisted objective procedures, the following well-established methods of measuring were applied: (1) maximum phonation time (MPT) as a statement of aerodynamic capacity [22, 23], (2) jitter as a common perturbation measure in acoustic analysis to assess frequency instability [24, 25], and (3) VRP to display the functional interactions of different voice production components with regard to vocal frequency and intensity [26, 27]. Recordings were performed in the sound-treated (one room) voice lab of our department with a background noise < 40 dB (A), using the DiVAS software (XION Medical, Berlin, Germany). A head-mounted microphone with a stable mouth-microphone distance of 30 cm was applied. Relevant instructions of individuals performing objective acoustic measurements are given in Table 1.

The detailed procedure of VRP measurement is described in a previous publication [7]. The parameters' highest tone (F_0_high), lowest intensity (SPLmin), MPT, and jitter were used for DSI calculation [9]. The Gonnermann-classification served for DSI interpretation, discriminating healthy voices (≥4.2) from mildly (<4.2 to ≥1.8), moderately (<1.8 to ≥-1.2), or severely (<-1.2) dysphonic voices [28]. VEM calculation was conducted automatically after VRP recording by the proprietary AVA software [8, 13].

### 2.4. Subjective Vocal Impairment and Voice Usage

To take the subject's self-assessment into account, every study participant had to fill out the VHI-9i questionnaire [29]. This item-reduced VHI consists of nine questions addressing functional, physical, and emotional impairments, describing the impact of a voice disorder on a person's quality of life. Each question can be rated on a scale from 0 to 4 (0: never; 1: almost never; 2: sometimes; 3: almost always; 4: always). The VHI-9i total score ranges from 0 to 36 and allows an impairment-related severity classification (0-5: no dysphonia; 6-13: mild dysphonia; 14-22: moderate dysphonia; 23-36: severe dysphonia) as proposed by Seipelt and Nawka [30]. Furthermore, the questionnaire registers the subject's profession as well as the individual use of speaking and singing voice. Simplifying the vocal use classification system by Koufman and Isaacson [31], the cohort was divided into “professional voice users” (PVU) and “non-professional voice users” (nPVU) depending on the reported occupation and voice usage.

### 2.5. Statistical Analysis

All data were statistically analyzed using SPSS version 25 (IBM Corp., Armonk, N.Y., USA). Descriptive statistics were applied to evaluate the basic quantitative features of the data collected. We documented the minimum and maximum values, calculated medians, first and third quartiles, and means and standard deviations (SD) for all objective and subjective parameters as well as patients' sociodemographic data. By using the one-way analysis of variance (ANOVA) with post hoc analysis, we examined the differences of mean values and their significance considering pathology subgroups, gender, and voice usage. Scatterplots with regression lines were made for females and males to display the dependence of VEM and other objective parameters on VHI-9i. Boxplots were chosen as graphical technique to present pathology-related VEM and DSI ranges. Pearson's correlation coefficients were computed to investigate the strength and direction of association between the individual parameters in all study participants and in each pathology subgroup. The level of significance was set at *α* = .05. The following abbreviations were used to show different significance levels: ^∗^ = 5%; ^∗∗^ = 1%; ^∗∗∗^ = 0.1%.

## 3. Results

### 3.1. Description of the Study Cohort

**Figure 1:**
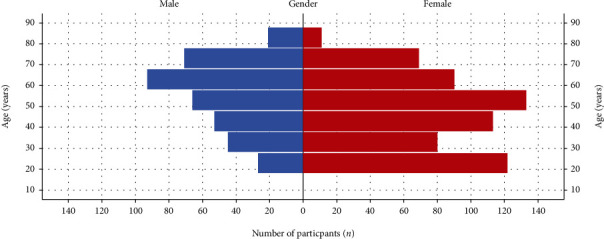
Histograms of age by gender in the total cohort.

The complete dataset comprised 994 study participants, 376 male (18-86 years, median 57) and 618 female subjects (18-82 years, median 47). Women were on average 9 years younger than men at the time of consultation (46 ± 17 vs. 55 ± 16, mean ± SD, and *p* < 0.05). The age and gender distribution of the examined cohort is shown in Figure 1.

VLS revealed in 691 subject (69.5%) organic diseases at VF level. Classification of the resulting structural dysphonia according to the underlying pathology showed in 26.5% diseases of the lamina propria (e.g., nodules, polyps, cysts, and edema), in 15.9% neurogenic voice disorders (e.g., VF paralysis and spasmodic dysphonia), in 14.7% diseases of the epithelium (e.g., leukoplakia, hyperkeratosis, carcinoma, and papillomatosis), and in 12.4% inflammatory findings (e.g., laryngitis). Additionally, VLS did not show any organic changes in 303 study participants (30.5%). From those individuals with normal laryngeal anatomy, 22.5% suffered from a vocal load-induced functional dysphonia, while 8.0% were completely healthy without any voice complaints (control group). Comparing our pathology subgroups, most voice disorders occurred in the 5th decade of life, except lamina propria changes (4th decade) and malignant lesions (5th to 6th decade). A detailed description of the investigated parameters in our study population is presented in Table 2.

### 3.2. Effects of Gender, Voice Use, and Age

In both genders, the overall vocal, aerodynamic, and psychometric parameters indicated mild to moderate impairments. Males and females had mildly dysphonic voices with slightly reduced vocal capacities. According to the mean VHI-9i scores (16 ± 8 vs. 15 ± 9), the self-assessed vocal impairment was comparable. Females revealed higher mean values for F_0_high and DSI, as well as lower values for SPLmin, jitter, and VHI-9i. Males reached higher values for MPT and VEM. These gender-specific differences were statistically significant for the parameters F_0_high, SPLmin, MPT, DSI (all *p* < 0.001), and jitter (*p* < 0.05), but not significant for VEM and VHI-9i.

Regarding voice usage, this cohort consisted of 3 groups: 29% PVU (*n* = 286, e.g., teachers, preschool teachers, lecturers, instructors, salespersons, singers, and actors), 59% nPVU (*n* = 585, e.g., administration employees, laborers, business (wo)men, pensioners, and unemployed people), and 12% with missing answers concerning occupation (*n* = 123). When consulting a voice specialist in our department, PVU were on average 8 years younger than nPVU (44 ± 14 vs. 52 ± 18 years, *p* < 0.001). The voice use had a significant influence on the objective parameters: compared to nPVU, PVU generally reached higher mean values for F_0_high, MPT, DSI, and VEM (all *p* < 0.001), as well as the lowest mean values for SPLmin (*p* < 0.001) and jitter (*p* < 0.05). However, comparison of the VHI-9i scores revealed that the subjective impairment did not differ significantly between PVU and nPVU.

**Figure 2:**
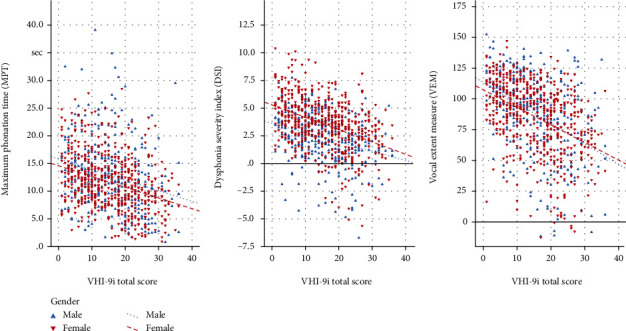
Scatterplots and regression lines of objective vocal parameters MPT, DSI, and VEM in relation to subjective impairment-related VHI-9i scores separated for males and females.

Focusing on VHI-9i results, 22.4% of all subjects felt highly (score 23-36), 33.2% moderately (score 14-22), and 30.5% mildly affected (score 6-13), while 13.9% of the participants did not indicate relevant voice impairment (score 0-5). However, subjective complaints rose with age. According to the severity classification, the mean age continuously increased as follows: 44 ± 19 (no dysphonia) vs. 46 ± 18 (mild dysphonia) vs. 52 ± 17 (moderate dysphonia) vs. 54 ± 14 years (severe dysphonia). These age-dependent VHI deteriorations were significant in women (*p* < 0.001) and showed a trend in men (*p* = 0.069). In accordance with rising subjective impairment levels, the objective parameters gradually worsened as well (i.e., constant decline of F_0_high, MPT, DSI, and VEM vs. increase of SPLmin and jitter). In both genders, these changes were highly significant (*p* < 0.001), apart from men's SPLmin (*p* < 0.05) and jitter (*p* = 0.075). Exemplarily, Figure 2 visualizes the scatterplots for the parameters MPT, DSI, and VEM on the *y*-axis related to VHI-9i on the *x*-axis.

### 3.3. Correlation Analysis and VEM Grading System

Correlation analysis showed that VEM correlated significantly (*p* < 0.01) with F_0_high (*r* = 0.48^∗∗^), SPLmin (*r* = −0.46^∗∗^), MPT (*r* = 0.44^∗∗^), jitter (vs. *r* = −0.15^∗∗^), and DSI (*r* = 0.63^∗∗^). DSI and VEM correlated furthermore with VHI-9i (*r* = −0.36^∗∗^ vs. *r* = −0.42^∗∗^). The strength of these relationships was moderate, apart from the weak negative relationship between VEM and jitter. Regarding age, correlations showed significant (*p* < 0.01) moderate relationships with F_0_high (*r* = −0.45^∗∗^), SPLmin (*r* = 0.32^∗∗^), and DSI (*r* = −0.44^∗∗^), compared to weak relationships with jitter (*r* = 0.12^∗∗^), VEM (*r* = −0.27^∗∗^), and VHI-9i (*r* = 0.21^∗∗^).

Finally, derived from the VEM percentiles of the total cohort (25%: 69; 50%: 93; 75%: 108), we created a grading system to compare the means of the investigated parameters on different VEM levels (Table 3). As a result, the higher the VEM, the higher the mean values for F_0_high, MPT, and DSI, and the lower the mean values for SPLmin, jitter, and VHI-9i. The VEM also seems to decrease as the voice ages. Regardless of the underlying pathology, subgroups I-VI comprised every VEM level. The mean values of all vocal parameters differed significantly between the VEM levels (*p* < 0.001).

### 3.4. Effects of Pathology in all Subgroups

Subgroup I contained patients with carcinoma in situ (*n* = 8), pT1a (*n* = 55), and pT1b (*n* = 12) squamous cell carcinoma. Males were significantly more often affected than females (average ratio 6.5: 1). At initial diagnosis, women were on average 12 years younger than men (*p* < 0.05). The aerodynamic and acoustic objective measurements confirmed the highly impaired voices in both genders. Compared to men, women had “better” mean values for all objective parameters apart from MPT and VEM. In contrast to that, their subjective suffering was higher (21 ± 6 vs. 17 ± 8), but not on a significant level (*p* = 0.165). In comparison with other pathology subgroups, mean values were the worst for F_0_high, SPLmin, jitter, DSI, and VEM.

Subgroup II involved the following pathologies: VF nodules (*n* = 91), Reinke's edema (*n* = 76), VF polyps (*n* = 33), sulcus vocalis (*n* = 22), VF cysts (*n* = 18), VF scars (*n* = 11), vascular malformation (*n* = 5), bamboo nodes (*n* = 4), and VF hematoma (*n* = 3). Females were significantly more often affected than males (average ratio 3.3: 1), especially concerning Reinke's edema (11.7: 1) and VF nodules (7.3: 1). Apart from the latter diagnoses, we found no relevant gender-specific differences for age, size, or location of findings at initial consultation. Women showed significantly higher values for F_0_high (*p* < 0.001) and lower values for MPT (*p* < 0.001) as well as VEM (*p* < 0.05). Gender-independent comparison between nodules, polyps, and Reinke's edema regarding mean VEM (101 ± 23 vs. 84 ± 24 vs. 72 ± 29) and DSI (4.4 ± 2.0 vs. 2.5 ± 1.7 vs. 2.0 ± 2.0) revealed highly significant differences (*p* < 0.001).

Subgroup III included patients with laryngeal papillomatosis (*n* = 38), leukoplakia (*n* = 20), and VF granuloma (*n* = 14). Men were more often affected than women (average ratio 1.6: 1) and on average 9 years older at first presentation (*p* < 0.05). Apart from the known differences regarding F_0_high (*p* < 0.001) and MPT (*p* < 0.05), both genders revealed similar results for SPLmin, jitter, and VHI-9i. However, higher values for VEM in males and DSI in females did not reach the level of significance.

Subgroup IV patients had acute (*n* = 26) or chronic laryngitis (*n* = 97). Females and males were nearly equally affected (average ratio 1.3: 1) and showed comparable means in age, SPLmin, jitter, and VHI-9i. Gender-specific differences were significant for F_0_high (*p* < 0.001), MPT, and VEM (*p* < 0.05), but not for DSI (*p* = 0.191).

Subgroup V involved the following heterogeneous pathologies: unilateral paralyses of the recurrent laryngeal nerve (*n* = 124), vagal nerve (*n* = 8), or external branch of the superior laryngeal nerve (*n* = 4), adductor spasmodic dysphonia (*n* = 19), Parkinson's disease (*n* = 2), and amyotrophic lateral sclerosis (*n* = 1). Females were more often affected than males (average ratio 1.7: 1). Both genders matched in age and presented with substantially impaired vocal parameters. SPLmin, MPT, jitter, and VHI-9i did not expose relevant gender-specific differences. Higher mean values reached the level of significance in women for F_0_high (*p* < 0.001) and DSI (*p* < 0.05), but not in men for VEM (*p* = 0.101). In comparison with other pathology subgroups, mean values were the worst for MPT and VHI-9i.

Subgroup VI included patients with dysodia (*n* = 119), hypo-/hyperfunctional dysphonia (*n* = 65), and presbyphonia (*n* = 34), as well as paradoxical VF movement disorder (*n* = 6). Females were more often affected than males (average ratio 1.8: 1) and on average 7 years younger when consulting the voice specialist (*p* < 0.05). Despite moderately impaired VHI-9i scores, both genders reached the highest values of all dysphonic patients for F_0_high, MPT, DSI, and VEM. Significant gender-specific differences were not detected, apart from F_0_high and DSI (*p* < 0.001). Gender-independent comparison between presbyphonic, dysodic, and hyperfunctional patients regarding mean VEM (78 ± 29 vs. 97 ± 24 vs. 107 ± 17) and DSI (2.0 ± 2.0 vs. 4.0 ± 2.2 vs. 5.5 ± 1.4) exposed highly significant differences (*p* < 0.001).

Subgroup VII consisted of considerably more women than men (average ratio 5.6: 1). All subjects showed normal laryngeal findings in the VLS and passed the vocal fitness examination. Compared to pathology subgroups, both genders presented at a much younger age and reached the best values in all observed vocal parameters (*p* < 0.001). Within this group, gender-specific differences showed significantly higher means for MPT, jitter, and VEM in men (all *p* < 0.05), as well as for F_0_high in women (*p* < 0.001).

**Figure 3:**
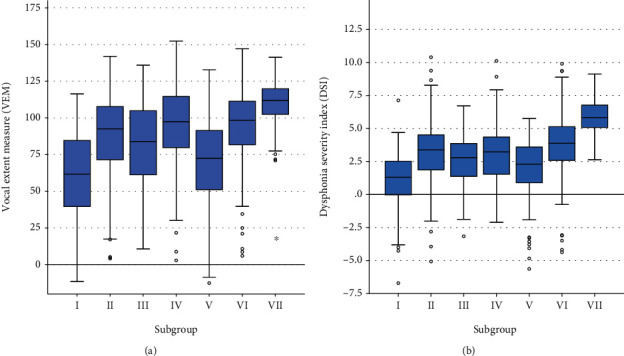
Comparison of (a) VEM and (b) DSI in each pathology subgroup. Median, quartiles, and range of values covered by the data, and any outliers (single spots) are presented using boxplots.

Comparison of all subgroups demonstrated that means for all vocal parameters differed significantly between them (*p* < 0.001). The post hoc analysis revealed that controls with a healthy voice were reliably distinguished from patients with an impaired voice in all examined parameters. Exemplarily, pathology-specific ranges for VEM and DSI are shown in Figure 3.

## 4. Discussion

In this study, we examined the pathology-induced changes of different voice disorders on the acoustic and aerodynamic parameters of the VRP with focus on the new VEM. Our results showed novel VEM outcomes concerning (1) variation between different pathology subgroups, (2) correlation with corresponding DSI values, (3) reflection of subjective voice impairment, and (4) changes with regard to voice usage, gender, and age differences. However, the heterogeneity of results regarding how pathology affected the investigated vocal measures and their relations must be critically discussed. From a clinical viewpoint, it is still not enough known about how these differences are explicable by vocal training, age, or gender and how to separate these factors from pathology.

### 4.1. Effects of Gender, Voice Use, and Age

The VEM exceeded the common range of 0 to 120 at both ends without showing any gender-specific differences (males: -12 to 152 [mean 88] vs. females: -13 to 147 [mean 85]). It correlated significantly with the DSI and corresponded to the same moderate subjective impairment level in men and women. The VEM showed a higher correlation with the VHI-9i total score than the DSI. Therefore, the VEM reflected the subjective perception better. Former studies stated that DSI and VHI are complementing parameters since there was no significant correlation detected [32, 33]. We conclude that VEM, DSI, and VHI represent complementing but different dimensions in voice diagnostics.

In contrast to VEM, a significant gender difference was found in F_0_high, SPLmin, MPT, and DSI. Females kept their physiologically higher F_0_high in all pathologies and in the healthy vocal state, resulting in a higher DSI (even when feeling more impaired). Other investigations confirmed a significantly higher F_0_high in females and the tendency for higher MPT in males, but could not detect a significant gender difference for DSI in subjects without voice complaints [34, 35]. Another study indicated that the DSI is influenced by the registration program as well as gender in patients with voice disorders [7]. The present investigation confirms these findings partially. When comparing the total cohort by gender, there was a significant difference found in DSI values, but in subgroup analysis, only patients with neurogenic lesions (V) and functional disorders (VI) differed on a significant level. Interestingly, those two groups are the ones where both genders showed no significant difference in MPT measures. Therefore, the influence of MPT as a considerable compensation mechanism in multidimensional DSI calculation must be further discussed.

Regarding VEM differences between PVU and nPVU, Caffier et al. [14] showed in patients with VF nodules a significant higher vocal capacity in PVU, with similar subjective VHI-9i impairment levels. Our study confirmed these results. Even among healthy subjects, a small proportion rated their voices mildly to moderately impaired according to the VHI-9i. All of them were female and prospective or active PVU. One explanation could be that PVU have particularly high demands and expectations regarding their vocal quality and performance. They depend on a proper function of their voices to “perform” at its best in their profession. When entering a vocal fitness examination, especially females tend to assess their voices overcritically, even when VRP measurements display a healthy or exceptional great voice. This is in line with the results of Chitguppi et al., which showed that female PVU with no obvious VF pathology reported significantly more voice-related complaints than nPVU, while reaching in all investigated objective vocal parameter values above average [36].

As expected, younger age was associated with higher VEM values. Similar to former studies, the age distribution revealed the majority of our patients in the working population, where the voice is most needed [37–39]. The age difference of 8 years implies that PVU consult a physician at an earlier stage, but we did not measure the exact pathology sizes in the VLS to prove this assumption. The VEM was highest in young healthy subjects. The VEM quartiles showed a decrease of VEM with rising age. However, lifelong laryngeal training can postpone signs of vocal aging [40, 41]. Regardless of age, the presented values of VEM quartiles (Q1: VEM < 69; Q2: VEM ≥ 69 to <93; Q3: VEM ≥ 93 to <108; Q4: VEM ≥ 108) could serve in the future as a reference range for the classification of vocal capacity in dysphonic patients and healthy subjects.

### 4.2. Effects of Pathology in all Subgroups

The VEM in all pathology subgroups differed significantly, without resulting in specific values or ranges that could (pre)determine the medical diagnoses. Every pathology influenced the intensity and frequency range on a distinct level, so that the VEM varied in one pathology from slightly to highly reduced. As known, the VRP aids to present the patients phonatory status without predicting the diagnosis [42]. Other studies confirmed that the VRP contour and its parameters are influenced by an organic voice disorder without showing a pathology-specific VRP configuration [43, 44].

Similar to previous studies, healthy subjects (VII) reached the highest VEM and DSI values while assessing their voices being not impaired [7, 16]. They could be clearly separated from patients with voice disorders by VEM and all other examined parameters. Subjects diagnosed with functional voice disorders (VI) had the best VEM and DSI values of the dysphonic patients and assessed their voices as moderately impaired. Dysfunction without organic changes is accompanied by impaired aerodynamic and acoustic measures [45–47]. Within the heterogenic group of functional dysphonia, we found significant VEM differences between presbyphonic, dysodic, and hyperfunctional patients, proposing that the term “non-organic/functional” voice disorder is widely composed and needs clarification in nomenclature settings.

Patients with malignant epithelial lesions (I) had the lowest VEM and can be clearly separated from other subgroups. The VEM also differed significantly from benign changes of the epithelium (III). Likewise, Kang et al. [43] detected a lower objective voice quality (jitter, shimmer, and noise-to-harmonic ratio) as well as a higher subjective impairment (VHI) in patients with glottic carcinoma compared to benign epithelial lesions. The change of the microarchitecture of the epithelial layer leads to an irregular rough surface and voice abnormalities due to increased mass, interference with vibratory behavior, and compromised VF closure. Confirming the literature, increasing thickening and infiltration resulted in a reduced or absent mucosal wave with growing stiffness of the lesion; the glottal closure could be also progressively impaired [48–50].

Patients with neurogenic voice disorders (V) assessed their voices higher impaired than the carcinoma group (I), but showed better functional measurements (except for MPT). This heterogeneous subgroup involved absent, decreased, or increased VF motions due to neurological or muscular disorders causing glottal insufficiency or dystonic spasms in varying degrees. As known from other studies, patients with unilateral VF paralysis reported vocal fatigue, dys-/aphonia and a breathy voice due to air escape; the extent depends on the position of the paralyzed VF [51, 52]. Omori et al. [53] found the glottal gap size as the main factor for reduced voice quality rather than the underlying diagnosis. Therefore, the comparable VEM in neurogenic and malignant lesions can be partly explained by the amount of glottal insufficiency, depending on the extent of infiltration, respectively, paralysis.

Benign changes of the lamina propria (II), the epithelium (III), and inflammatory lesions (IV) led to comparable moderately impaired VEM, DSI, and VHI-9i values. As expected, all included pathologies interfered with the vibratory behavior and closure pattern and resulted in roughness and/or breathiness. Confirming previous studies, more diffuse lesions with an excess of liquid, gelatinous, or fibrous material raised the VF stiffness and increased (e.g., Reinke's edema) or decreased (e.g., laryngitis and sulcus vocalis) the mucosal wave [21, 54]. On the other hand, the VEM was able to differentiate between VF nodules, polyps, and Reinke's edema by showing a different vocal capacity related to the pathology dimensions.

### 4.3. Limitations

This study involves a few limitations, which should be considered when drawing general conclusions. Firstly, auditory perceptual assessment was unfortunately not included in the initial study design. Due to the large amount of data, a retrospective blinded voice evaluation of 994 patients with 4-5 raters was not an available option. Secondly, the exact extent of the leading pathology was not classified or measured. However, the importance of the size of the findings should not be underestimated. For example, Salmen et al. found in patients with varying sizes of Reinke's edema significant differences in VEM and DSI values depending on the Yonekawa classification [15]. Thirdly, various subgroups (e.g., II and VI) were very heterogeneous in itself, creating the false impression that the vocal capacity, respectively, impairments of different pathologies included are alike. Fourthly, the effects of age on the VEM can only be estimated since our patients above 45 years were all classified with a voice disorder. Considering the close relationship to the DSI and its proven influence on age [34, 35, 55], an age effect is likely. As there is no reliable differentiation between the effects of age and pathology, further investigations should include a larger age variety of healthy subjects. Besides, the VEM can only be calculated on the basis of a completed VRP. However, due to the parameter construction, an aphonic voice cannot be quantified by the VEM. In addition, further studies are necessary relating the presented VEM quartiles (Q1: VEM < 69; Q2: VEM ≥ 69 to <93; Q3: VEM ≥ 93 to <108; Q4: VEM ≥ 108) to the auditory perceptual assessment of voices.

## 5. Conclusions

We conclude that the VEM proved to be a comprehensible and easy-to-use interval-scaled parameter for objective VRP evaluation in all laryngeal pathologies. By quantifying the vocal capacity, it represents vocal function as a positive measure and should therefore be established as a useful and complementary parameter in addition to the DSI. Besides, the VEM seems to reflect the subjective impairment better than the traditional DSI. However, the size of the VEM value alone does not allow to draw conclusions about the underlying diagnosis.

## Figures and Tables

**Table 1:** Instruction of individuals performing objective acoustic measurements.

Measure	Phonatory tasks
Jitter	Standing position. Produce a sustained vowel (/na/or/a/) for 3-5 seconds. Use a comfortable pitch and loudness. *The most stable recording out of 3 trials was chosen for DSI calculation.*

MPT	Standing position. Produce a sustained vowel (/a/or/na/) for as long as possible after maximum inhalation. Use a comfortable pitch and loudness. *The longest phonation time out of 3 trials was chosen for DSI calculation.*

VRP	Standing position. Initial assessment of speaking VRP, followed by singing VRP. (1) Speaking voice. Count from 21 to 25 at different intensity levels 1a: Lowest volume possible (unstressed phonation at indifferent pitch) 1b: Normal conversation volume (fist level of increase) 1c: Speaker's voice (lecture hall volume, second level of increase) 1d: Loudest volume possible (calling voice) (2) Singing voice. Produce a sustained vowel (/na/or/a/) for at least 2 seconds at different intensity levels 2a: As softly as possible 2b: As loud as possible For both settings, begin at a comfortable middle pitch. Stepwise reduce the pitch to the lowest possible note. Return to comfortable pitch and get stepwise higher in pitch as far as possible. *The perimeter and area between the curves of soft and loud singing VRP served for VEM calculation.*

**Table 2:** Characteristics of vocal parameters considering gender, age, and pathology subgroups. The number (*n*) and percentage (%) of participants, as well as the mean values and standard deviations (mean ± SD) for age (in years), highest frequency (F_0_high in hertz), lowest intensity (SPLmin in decibel), maximum phonation time (MPT in seconds), jitter (in %), dysphonia severity index (DSI), vocal extent measure (VEM), and voice handicap index (VHI-9i) are displayed.

	Cohort I-VII	Subgroup I	Subgroup II	Subgroup III	Subgroup IV	Subgroup V	Subgroup VI	Subgroup VII
Participants (number, %)	994 (100%)	75 (7.5%)	263 (26.5%)	72 (7.2%)	123 (12.4%)	158 (15.9%)	224 (22.5%)	79 (8.0%)
Male	376 (37.8%)	65 (86.7%)	61 (23.2%)	44 (61.1%)	54 (43.9%)	59 (37.3%)	81 (36.2%)	12 (15.2%)
Female	618 (62.2%)	10 (13.3%)	202 (76.8%)	28 (38.9%)	69 (56.1%)	99 (62.7%)	143 (63.8%)	67 (84.8%)

Age (years, mean ± SD)	49 ± 17	62 ± 13	44 ± 15	54 ± 15	53 ± 14	56 ± 14	52 ± 18	25 ± 9
Male	55 ± 16	64 ± 12	45 ± 15	58 ± 14	53 ± 15	57 ± 13	57 ± 18	23 ± 4
Female	46 ± 17	52 ± 14	43 ± 14	49 ± 16	52 ± 14	55 ± 14	50 ± 17	25 ± 9

F_0_high (Hz, mean ± SD)	470 ± 188	324 ± 112	463 ± 180	388 ± 130	458 ± 198	413 ± 138	518 ± 176	709 ± 173
Male	355 ± 116	309 ± 99	372 ± 134	325 ± 104	382 ± 130	318 ± 90	384 ± 97	507 ± 110
Female	540 ± 190	419 ± 144	490 ± 182	488 ± 102	517 ± 220	470 ± 130	594 ± 165	742 ± 158

SPLmin (dB, mean ± SD)	50 ± 5	53 ± 5	49 ± 4	51 ± 5	51 ± 5	51 ± 5	49 ± 5	47 ± 3
Male	51 ± 5	53 ± 5	50 ± 5	51 ± 5	52 ± 5	51 ± 5	50 ± 6	47 ± 3
Female	49 ± 4	51 ± 5	49 ± 4	51 ± 5	51 ± 5	50 ± 4	49 ± 4	47 ± 3

MPT (s, mean ± SD)	12 ± 6	11 ± 5	12 ± 6	13 ± 6	13 ± 6	9 ± 5	14 ± 5	16 ± 5
Male	13 ± 7	11 ± 5	14 ± 7	14 ± 6	14 ± 6	10 ± 7	15 ± 6	20 ± 7
Female	12 ± 5	10 ± 5	11 ± 5	10 ± 4	12 ± 5	9 ± 4	13 ± 5	15 ± 4

Jitter (%, mean ± SD)	0.3 ± 0.8	0.7 ± 1.2	0.3 ± 0.7	0.3 ± 0.5	0.2 ± 0.1	0.6 ± 1.1	0.3 ± 0.8	0.1 ± 0.1
Male	0.4 ± 0.9	0.7 ± 1.3	0.3 ± 0.4	0.3 ± 0.6	0.2 ± 0.1	0.6 ± 1.1	0.4 ± 0.9	0.2 ± 0.1
Female	0.3 ± 0.7	0.3 ± 0.6	0.3 ± 0.8	0.2 ± 0.1	0.2 ± 0.1	0.5 ± 1.1	0.3 ± 0.8	0.1 ± 0.1

DSI (mean ± SD)	3.1 ± 2.4	1.1 ± 2.3	3.2 ± 2.2	2.6 ± 1.9	3.0 ± 2.2	2.0 ± 2.2	3.8 ± 2.2	5.9 ± 1.4
Male	2.4 ± 2.3	0.8 ± 2.2	3.0 ± 1.8	2.4 ± 2.2	2.7 ± 2.1	1.5 ± 2.2	3.1 ± 2.0	5.4 ± 1.6
Female	3.6 ± 2.4	2.6 ± 2.5	3.3 ± 2.3	2.9 ± 1.4	3.2 ± 2.3	2.3 ± 2.1	4.2 ± 2.2	6.0 ± 1.3

VEM (mean ± SD)	86 ± 31	60 ± 31	88 ± 28	81 ± 30	93 ± 30	70 ± 31	95 ± 25	109 ± 18
Male	88 ± 33	61 ± 30	96 ± 28	86 ± 32	102 ± 27	76 ± 32	99 ± 28	121 ± 13
Female	85 ± 30	50 ± 39	86 ± 28	75 ± 26	87 ± 30	67 ± 30	92 ± 24	106 ± 18

VHI-9i (mean ± SD)	15 ± 9	18 ± 8	15 ± 8	15 ± 9	14 ± 8	21 ± 9	14 ± 8	6 ± 5
Male	16 ± 8	17 ± 8	15 ± 8	15 ± 9	14 ± 7	20 ± 9	15 ± 8	4 ± 1
Female	15 ± 9	21 ± 6	15 ± 8	14 ± 9	14 ± 9	22 ± 8	14 ± 8	7 ± 6

Subgroup I—malignant pathologies, subgroup II—lesions of the lamina propria, subgroup III—benign changes of the epithelium, subgroup IV—inflammatory changes, subgroup V—neurogenic voice disorders, subgroup VI—functional disorders, and subgroup VII—healthy control group.

**Table 3:** Effects of VEM levels on the investigated parameters (mean ± SD).

VEM level	Participants *n* (%)	Age (years)	F0high (Hz)	SPLmin (dB)	MPT (s)	Jitter (%)	DSI	VEM	VHI
0	243 (24.4%)	40 ± 17	606 ± 203	47 ± 3	15 ± 5	0.2 ± 0.2	5.2 ± 1.7	120 ± 9	11 ± 7
1	241 (24.2%)	49 ± 18	501 ± 170	49 ± 4	14 ± 5	0.3 ± 0.9	3.7 ± 2.0	101 ± 4	14 ± 7
2	252 (25.4%)	52 ± 17	431 ± 148	50 ± 4	11 ± 5	0.3 ± 0.7	2.8 ± 1.6	82 ± 7	16 ± 9
3	258 (26.0%)	55 ± 14	352 ± 127	53 ± 5	9 ± 5	0.5 ± 1.1	1.0 ± 2.1	44 ± 20	21 ± 7

The grading of VEM levels was based on the percentiles of the total study cohort (*n* = 994). Level 0: normal vocal capacity (VEM ≥ 108) ≙ 100th percentile (Q4). Level 1: mildly reduced vocal capacity (VEM < 108 to ≥93) ≙ 75th percentile (Q3). Level 2: moderately reduced vocal capacity (VEM < 93 to ≥69) ≙ 50th percentile (Q2). Level 3: severely reduced vocal capacity (VEM < 69) ≙ 25th percentile (Q1).

## Data Availability

The datasets produced and investigated for this study are not publicly available, as they were obtained from a proprietary database via a licensing agreement.
